# Methodological limitations of psychosocial interventions in patients with an implantable cardioverter-defibrillator (ICD) A systematic review

**DOI:** 10.1186/1471-2261-9-56

**Published:** 2009-12-29

**Authors:** Elena Salmoirago-Blotcher, Ira S Ockene

**Affiliations:** 1 University of Massachusetts Medical School, 55 Lake Ave Worcester, MA 01655-0002, USA

## Abstract

**Background:**

Despite the potentially life-saving benefits of the implantable cardioverter-defibrillator (ICD), a significant group of patients experiences emotional distress after ICD implantation. Different psychosocial interventions have been employed to improve this condition, but previous reviews have suggested that methodological issues may limit the validity of such interventions. Aim: To review the methodology of previously published studies of psychosocial interventions in ICD patients, according to CONSORT statement guidelines for non-pharmacological interventions, and provide recommendations for future research.

**Methods:**

We electronically searched the PubMed, PsycInfo and Cochrane databases. To be included, studies needed to be published in a peer-reviewed journal between 1980 and 2008, to involve a human population aged 18+ years and to have an experimental design.

**Results:**

Twelve studies met the eligibility criteria. Samples were generally small. Interventions were very heterogeneous; most studies used cognitive behavioural therapy (CBT) and exercise programs either as unique interventions or as part of a multi-component program. Overall, studies showed a favourable effect on anxiety (6/9) and depression (4/8). CBT appeared to be the most effective intervention. There was no effect on the number of shocks and arrhythmic events, probably because studies were not powered to detect such an effect. Physical functioning improved in the three studies evaluating this outcome. Lack of information about the indication for ICD implantation (primary vs. secondary prevention), limited or no information regarding use of anti-arrhythmic (9/12) and psychotropic (10/12) treatment, lack of assessments of providers' treatment fidelity (12/12) and patients' adherence to the intervention (11/12) were the most common methodological limitations.

**Conclusions:**

Overall, this review supports preliminary evidence of a positive effect of psychosocial interventions on anxiety and physical functioning in ICD patients. However, these initial findings must be interpreted cautiously because of important methodological limitations. Future studies should be designed as large RCTs, whose design takes into account the specific challenges associated with the evaluation of behavioural interventions.

## Background

Implantable cardioverter-defibrillators (ICD)[1] are electronic devices used to prevent sudden cardiac death and to treat severe ventricular arrhythmias. Primary and secondary prevention trials have consistently shown that ICDs reduce the risk of cardiac death [2-6], making them the first-choice treatment for patients at risk of sudden cardiac death. Since the approval of ICDs by the Food and Drug Administration in 1985, the number of ICD implantations performed in the United States has steadily increased. The estimated number of hospitalizations for ICD implantation increased from 5,600 in 1990 to 108,680 in 2005, and the estimated annual rate of hospitalizations increased tenfold [7].

Despite the proven efficacy of ICDs, concerns have been raised regarding quality of life and psychological well-being of recipients of an ICD [8,9]. There are a number of stressors that can cause significant psychological discomfort [10] and consequently affect the quality of life of an ICD recipient. A substantial amount of anxiety is related to the shocks: patients are afraid of the pain caused by a shock[11] and of the circumstances related to the unpredictability of its occurrence, such as the possibility of receiving a shock while out of the house, or the reaction of bystanders not familiar with the patient or the ICD[12,13]. A further source of concern is the possible failure of the ICD, including concern that the system will not be able to control the dysrhythmia or concern about depending upon an electronic device for survival[13] Although many ICD patients adapt to the device over time, some degree of anxiety is experienced by 24% to 87% of patients, and a significant proportion (13% to 38%) experience symptoms compatible with a diagnosis of anxiety disorder [9].

Anxiety and depression have been shown to be independent predictors of mortality in ischemic heart disease [14-20], raising the possibility that anxiety plays a contributing role in the high one-year mortality rate observed after ICD implantation [6] despite the effectiveness of the ICD in preventing sudden death. Paradoxically, ICD patients might be at higher risk of having arrhythmias, and therefore of receiving shocks, because of their fear of receiving shocks. The role of anxiety and stress in inducing ventricular arrhythmias has been hypothesized since the 1970s [21,22] and in the 1990s evidence indicated that strong emotions can precipitate cardiac events [23-25]. Emotional [26,27] and mental stress[28] were shown to have a detrimental effect on both cardiac perfusion and function. This suggests that, at least in some settings, negative emotions may play a causal role in cardiac events, rather than being secondary phenomena. Anxiety may worsen cardiac outcomes by reducing heart rate variability (HRV) [29,30] and baro-reflex control[31] or by inducing alterations in the coagulation system [32]. Conversely, conditions promoting psychological well-being such as social support or pet ownership [33,34] may favourably influence variables such as HRV and survival.

Considering the negative impact of anxiety and depression on cardiac outcomes, over the past ten years considerable effort has been invested in designing interventions that can improve psychological wellbeing in ICD recipients. Preliminary evidence suggests that these interventions may be effective; however, most literature reviews, including recent ones [35-37] did not address many methodological issues related to the non-pharmacological nature of the intervention(s).

The aims of this review was to examine the methodology of previously published studies of psychosocial interventions in patients receiving an ICD, using the CONSORT[38] statement for non-pharmacological interventions as a guideline, and to provide recommendations for future research.

## Methods

We performed an electronic search of the databases PubMed, Cochrane, and PsychInfo using combinations of these terms: ICD, implantable cardioverter defibrillator, cardioverter defibrillator, implantable defibrillator, defibrillator, automatic defibrillator, intervention, treatment, rehabilitation, therapy, psychological, psychosocial, behavioural, depression, depressive disorder, anxiety, anxiety disorder, and quality of life.

To be included studies had to meet these criteria: 1) publication in a peer-reviewed journal after 1980, 2) include adult humans (≥18 years old), and 3) have an experimental design. Criterion 1 was chosen since the first ICD was implanted in 1980. Randomized clinical trials (RCT), quasi-randomized, and quasi-experimental studies were included.

We defined "psycho-social" as any non-pharmacological intervention aimed at improving the psychological well-being of ICD patients. Thus, our sample included studies evaluating interventions that were not strictly psychological, such as educational interventions, support groups, and cardiac rehabilitation. Studies evaluating the effect of mixed interventions (i.e., psychological intervention or support group combined with an exercise program) were also included. Literature reviews, case reports, and observational studies were excluded. In addition to the electronic search, reference lists of included articles and literature reviews were hand searched. The first author conducted the computer search; after eliminating double hits, both authors reviewed the abstracts for eligibility. We resolved any disagreement until we reached consensus.

An original data abstraction form was designed to collect information relevant to this review, using the CONSORT Statement Extension for Trials Assessing Non-pharmacologic Treatments [39,40] as a guideline. Due to the heterogeneous nature of the interventions and of the populations studied, a meta-analysis was not conducted.

## Results

### Study selection

**Figure 1:**
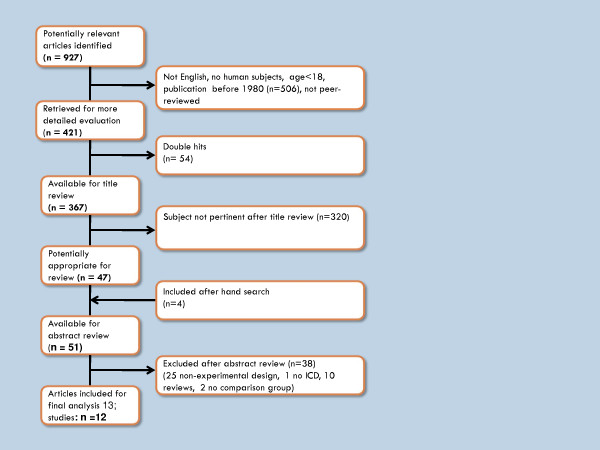
**Flow-chart of literature selection process**.

Our search initially identified 927 titles: 583 from PubMed, 222 from Cochrane, and 122 from PsychInfo. After applying the inclusion criteria, 506 titles were excluded. Removing 54 double hits left 367 studies. After title review, 320 articles were excluded because the study topic was not pertinent to this review, leaving 47 articles for abstract review. Another four articles were included after hand searching the reference lists of literature reviews. The literature selection process is summarized in Figure 1. Of the 51 articles whose abstracts were reviewed, 38 were excluded: 25 had a non-experimental design, one involved cardiac arrest survivors who did not receive an ICD, ten were literature reviews or editorials, and two lacked a comparison group.

The sample for review included 13 papers, of which two [41,42] reported on the same sample at different follow-up times and were considered one study. Twelve studies were thus included in this analysis (Additional File 1).

In this section, we will describe the included studies, summarize the population characteristics, and review the study interventions. We will then briefly describe the main statistical methods used, and summarize the intervention effects on psychological and cardiac outcomes.

### Characteristics of the included studies

#### Study design

Of the 12 studies reviewed, nine adopted a randomized controlled trial (RCT) design [41-50]; one study used a cluster RCT design [48], and two adopted a cross-over RCT design [46,47] (Additional File 1). In one study, patients were randomized before being asked to participate [51]. In the two non-randomized studies [52,53], the comparison group included patients unable to participate due to lack of transportation or illness.

Recruiting centres were teaching hospitals [43-45,49-52], or tertiary referral centres [41,46-48,53]. Recruitment duration was not reported in seven studies [44-47,49,52,53]; in the remaining studies [41,43,48,50,51] it ranged from 10 to 23 months. Recruitment information was often incomplete: some studies did not specify eligibility criteria [43,51,52], and others did not report the number of screened and/or eligible patients [41,43,45-47,49-53]. In the three studies specifying the number of eligible subjects, the proportions of patients who refused or were unable to participate were 15% [48], 65% [44], and 46% [47]. Lack of transportation or distance from study site was the most frequent reason for not being able to participate [45,47,52]. A flow diagram describing the progress of participants through the different phases of a RCT as recommended by the CONSORT statement [54] was included only in three studies [44,47,48].

#### Sample Size

Sample sizes were small (range 12-192) in most studies; in seven, the intervention or control arm had less than 20 participants.

#### Follow-up period

The duration of follow up varied from 1 to 12 months. In fifty percent of the studies, the follow up period was between 3 and 6 months.

#### Retention rates

Seven studies reported the number of dropouts (Additional File 1). One-year retention rates ranged from 57% [44] to 89% [42]; the study reporting the highest retention rate offered monetary compensation to participants. In studies with shorter (3-6 months) follow-ups, retention rates varied from 66% to 95%.

### Study Samples

Study samples (Additional File 2) were predominantly male (65% to 91%). In the four studies reporting racial distribution [41,48,49,51], Caucasians were the most highly represented group (77% to 98%). Most studies excluded patients younger than 18 or 21; the mean age varied from 58 to 66 years (range 28-83). Six studies enrolled only new ICD recipients [41,43,45,48,50,51]; one included both new and old recipients [44], and five enrolled old recipients only [46,47,49,52,53]. In the studies enrolling old recipients, mean time since initial implantation varied considerably, from 8 months (12.5 in the intervention group) [52] to 20 months (range 7-53) [46]. One study reported that 55% and 45% of participants were enrolled 5-6 months and 12-24 months post-ICD, respectively (52) and in two studies (38, 48) time since implantation was not reported.

The indication for ICD implantation or the underlying cardiac disease was not reported in most studies. The most common cardiac diagnosis was coronary artery disease (up to 69% of the population) [41,44,46,48,49]. Details about the type of ICD implanted were also missing; in one study [50] 50% of study participants had their ICD implanted using thoracotomy, a more invasive procedure than that currently used. Other studies [52,53] conducted during the same period (late 1980s-early 1990s) do not report the use of thoracotomy, but they might have included such patients. Mean ejection fraction (EF), when reported, was between 30% and 44% [41,44,46,50,52]. Information about New York Heart Association (NYHA) functional class was provided in two studies only: in one study the mean NYHA class was 1.6 and 2.0 in the intervention and control groups, respectively; in the second study [48] 75% of subjects were in NYHA class I or II. Collecting data on these variables both at baseline and during follow-up, and adjusting for them in the analysis is important because a worsening of underlying cardiac function can affect study outcomes. Similarly, information about important confounders such as the concurrent use of anti-arrhythmic and psychotropic drugs was not available in most studies. However, 15% to 20% of patients were reported to have received psychotropic drugs during two studies [49,48].

### Interventions

Details about the characteristics of the interventions are reported in Additional File 3. Interventions were extremely diverse; some studies used multi-component interventions [46-50], and others employed mono-component interventions (support groups [52,53], phone support [41], cognitive-behavioural (CBT) therapy [44,51], or educational interventions [43,45]. CBT was a core intervention component in six studies [44,46-49,51]. CBT was self-administered in one study [48], and specialist-delivered as a group intervention in the other studies. Kohn [51] employed one-to-one meetings; in another study [46] one-to-one CBT was available only to patients with high levels of anxiety.

In most studies, interventions were supplemented with relaxation techniques taught during sessions or self-taught by tape [44,46-48,50]. In three studies [46-48], patients were also involved in an aerobic exercise program as part of a multi-component intervention. The duration of interventions ranged widely, from 2 [45] to 20 weeks [51]; it was not reported in three studies[43,48,53]

The control condition was represented as "usual care" in eight studies, was not reported in two studies[52,53], and was described as "no therapy" in another [51]. Sears et al. reported delivering a shorter version of the intervention (one-day workshop in addition to usual care for ethical reasons [49]. Only eight studies reported what "usual care" entailed (48, 50, 52-54, 56-58); patients receiving "usual care" were exposed to very different procedures (Additional File 3).

Significant others were involved either as co-participants or because they were invited to attend study sessions in seven studies [43,45,46,50-53].

No study provided details about standardization of intervention delivery, i.e., attached scripts of the phone conversations or support meetings. As in most behavioural interventions, blinding of participants was impossible; attempts to blind outcome assessors or care providers not involved in the study were not mentioned. Information about adherence to study interventions, such as attendance at meetings or support groups, was not available except in Badger [52] (attendance at study meetings of 87.5%), while no study assessed treatment fidelity.

### Statistical analyses

In most studies, primary outcomes were not clearly specified. Of the ten studies using a RCT design, only three [41,44,48] used an "intention to treat" approach, of which one was a "modified" intention to treat [41] and one was used only for the primary outcome [44]. Repeated-measures ANOVA was used in most studies, and psychological outcomes were usually analyzed as continuous variables. Most studies reported no baseline differences between intervention and control groups for major demographic and co-morbidity covariates, but sample sizes were small. Only three studies [41,44,48] specified whether the final model was adjusted for covariates such as age, baseline outcome measures, use of psychotropic and anti-arrhythmic medications, EF and NYHA class, and time from ICD implantation. Power calculations were reported in three studies only [41,44,48].

### Psychological Outcomes

The effect of the intervention on anxiety [41,42,44-49,51,53] and depression [41,42,44-49,51] was assessed in all but three [50,52] studies (Additional File 4), and it was measured by self-administered questionnaires. Anxiety was most frequently measured by the Hospital Anxiety and Depression Scale (HADS) [46-48], and the State-Trait Anxiety Inventory (STAI);[41,49,51,53]. Depression was most often measured by the Beck Depression Inventory (BDI), [44,51] the Center for Epidemiological Studies Depression Scale (CES-D),[41,49] and the HADS [46-48]. Although studies sometimes used the same instrument, different outcomes were often evaluated. For example, some studies compared mean pre/post intervention scores within groups [41,46,53], while others compared mean pre/post intervention changes in HADS anxiety scores between groups [47], mean STAI anxiety scores between groups [51], or the change in the proportions of patients with HADS scores indicating significant anxiety [48]. Kohn [51] evaluated both STAI trait-anxiety and state-anxiety scores, while others [41,49,53] focused on state anxiety only.

#### Anxiety

Details about the impact of interventions on anxiety are presented in Additional File 4. Overall, of nine studies evaluating the impact on anxiety, six [44,46-49,51] showed a significant positive effect, while three [42,45,53] showed no improvement. In the two studies that measured psychological outcomes other than anxiety, one [52] reported no improvement in psychological adjustment, and another [50] reported no differences in the mood state profile at 4 months; both used a support-group intervention. All studies showing a positive effect included CBT either as the only intervention [51] or an element of a multi-component intervention [44,46-49]. The three studies showing no effect were either purely educational programs [45] or support interventions [41,53].

Biological markers of stress (salivary cortisol) and inflammatory markers (TNFα and IL-6) were included as outcome measures in one study (38). Salivary cortisol decreased significantly over time in both groups, while inflammatory markers did not change significantly.

#### Depression

Depression was a study outcome in eight studies, and only four ([46-48,51]) showed an effect. Fitchet ([46]) found a reduction in mean HADS depression scores from pre to post-rehabilitation in the intervention group (9.9 to 6.7 p < 0.001), while scores increased in the control group; in Frizelle study ([47]), HADS depression scores decreased significantly post vs. pre-treatment. Kohn ([51]) reported a decrease in BDI depression scores in the intervention group (6.9 ± 5.9 vs. 15.0 ± 13, but baseline depression scores were not reported. Lewin ([48]) found a reduction in the proportion of patients with HADS scores > 8 (probable significant clinical depression) in the intervention vs. the control group (-13% vs. -2.1%). Interestingly, of the four studies showing an effect on depression, three included an exercise component.([46-48])

#### Cardiac Outcomes

Half the studies (Additional File 5) included cardiac outcomes: shocks in six studies [41,44,46-48,51]; sustained ventricular tachycardia (VT) requiring pacing for termination in two [46,47], and ICD storms in one study [48]. Two studies [42,48] also evaluated the effect of the intervention on the number of hospitalizations and visits, and three [46-48] included exercise capacity. In the only study with HRV as an outcome [44], two indicators of autonomic tone improved, supporting the conclusion that the intervention improved adrenergic/vagal balance.

#### Shocks and heart rate variability

No study showed a significant effect on shocks. At 1 year of follow up, Chevalier [44] showed a non- significant reduction in the number of patients receiving a shock (3 in intervention vs. 6 in control group) and a reduction in shock rate and in use of beta-blockers and other anti-arrhythmic drugs in the intervention group (post-hoc analysis). Similarly, Lewin [48] showed a non-significant difference in the proportion of patients receiving shocks (9.5% vs. 13%) and ICD storms (1.6 vs. 4.8) at six months of follow-up. The mean number of shocks was similar in both groups.

#### Physical functioning

Physical functioning, measured as exercise capacity, improved significantly in three studies after a comprehensive cardiac rehabilitation (CCR) or ICD plan [46-48]. In one study [46], the pre- to post-intervention exercise time on a symptom-limited treadmill test increased 16% (p < 0.001) in the intervention group, but did not change in the usual care group. Frizelle et al. [47] found a significant improvement in level of difficulty (mean change pre-post intervention 1.37 vs. 0, p = 0.050) and the distance walked (mean change 85.6 vs. 0.32, p= 0.010) on a shuttle test. In another study [48], the Seattle angina scores of patients enrolled in the CCR improved significantly (difference in favour of intervention: 2.22, CI 0.11,7.22).

#### Hospitalizations

The two studies evaluating the impact on health care use yielded conflicting results. Dougherty [41,42] did not find any difference in the number of ER admissions, hospitalizations or clinic visits at 1 year, while Lewin [48] reported a significant reduction in the mean number of emergency admissions at 6 months (0.39 vs. 0.11, p = 0.05) and no differences in outpatients visits or routine admissions.

## Discussion

### Efficacy of the Interventions

Overall, the results of this review support previous, preliminary evidence [36] of a positive effect of psychosocial interventions in ICD patients. Two-thirds of the studies showed a favourable effect on anxiety, and half reduced depression. CBT seems to be the most effective intervention on anxiety, since all studies showing a positive effect included CBT either as the only intervention [51] or as an element of a multi-component intervention [44,46-49]. Three studies showing a positive effect on depression included an exercise component [46-48]. No evidence was found for a positive effect of psychosocial interventions on the number of shocks and arrhythmic events, probably due to the low frequency of shocks and short follow-up periods, and because most studies were not sufficiently powered to detect these outcomes. Only one study [44] documented an effect of the intervention (CBT) on heart rate variability; interestingly, the positive effect on adrenergic/vagal balance did not result in a reduction of arrhythmic episodes or shocks. Findings on physical functioning are promising, while only Lewin [48] showed an impact of the intervention on the use of health care facilities. Most studies used anxiety and depression scores as continuous variables, and generally showed a modest improvement in anxiety or depression scores, whose clinical significance has yet to be determined.

### Methodological limitations

Both positive and negative findings should be weighed against methodological limitations, which affected, to some degrees, most of the studies.

### Randomization

The purpose of randomization is to reduce selection bias, i.e. the uneven distribution of prognostic factors between the experimental group and the control group. Randomization reduces selection bias by creating comparison groups with similar characteristics regarding known and unknown confounders. Twenty-five percent of studies were not randomized or did not perform a correct randomization. Non-random allocation can lead to overestimation as well as underestimation of treatment effects[55].

### Study Samples

Study samples were generally small and heterogeneous. A small sample size limits the power to detect an effect, and increases the chances that important prognostic factors will differ between the intervention and the control group, even when randomization has been adequately performed.

### Measures

Psychological outcomes were determined by self-administered questionnaires, and an objective evaluation of the outcome was lacking. Most studies used anxiety and depression scores as continuous variables, and generally showed a modest improvement in anxiety or depression scores, whose clinical significance has yet to be determined. Some scales have cut-off scores for defining probably mild, moderate, or severe depression/anxiety[56]. Thus, the numbers of individuals with anxiety/depression scores above these cut-offs would be more clinically meaningful study outcomes [48].

### Interventions

Interventions were extremely different and even identical components (i.e., CBT) were administered under very diverse conditions across studies: for example, in some studies significant others were allowed to attend study sessions or were also enrolled, adding a possible support component. Relaxation techniques were used in most studies in adjunct to the "major" intervention(s), but very spare details were provided about these techniques, despite their potential to offer relief. In the case of multi-component interventions, it is impossible to determine which component was effective, to what extent, and if it had a cumulative effect. This issue is not merely academic, since multi-component interventions using different providers are more expensive than self-administered ones.

Finally, studies did not provide details about standardization of intervention delivery, i.e., attached scripts of the phone conversations or support meetings, and did not assess either patients' or providers' adherence to the intervention. As in most behavioural interventions, blinding of participants was impossible; attempts to blind outcome assessors or care providers not involved in the study were not mentioned.

### Comparison group

Most studies (8/12) used a "usual care" comparison group. According to the CONSORT[39] statement, information about the usual care group allows the readers to compare the experimental intervention with what is usually offered to patients as "regular" standard of care. Two out of eight studies did not provide details about what "usual care" would entail. When this information was available, what was offered as "usual care" differed greatly across studies (Additional File 3). This could have important consequences on the assessment of the difference between groups. Far from being "neutral", usual care assignment does expose patients to some form of "treatment": the more intense the treatment, the less the difference between the intervention and the control group. Furthermore, a "usual care" comparison group may not account for the additional attention received by the intervention group. An "attention control" or "active control" comparison group would be probably superior to usual care because it controls for nonspecific treatment effects that maybe associated with the intervention [57,58].

### Confounders

#### Device

Earlier studies enrolled patients who underwent thoracotomy and received more bulky devices. The implantation of such devices required a much longer and complex procedure than modern ICDs, possibly affecting pre-intervention anxiety levels.

#### Number of shocks and time from implantation

Some studies included both patients who had received previous shocks and those who had not. Psychological distress is higher in patients receiving shocks [59,60]. Device discharges may act as an effect modifier, i.e. the effect of the intervention may differ in patients who received or did not receive shocks. Also, patients were recruited at different times after ICD implantation, introducing the confounding factor of improved psychological adaptation to ICD over time[8]. This factor may explain the lack of effect among patients enrolled more than 1 year after ICD implantation [52,53].

#### Clinical characteristics

Data about the participants' clinical condition (e.g. NYHA class) were not reported or were reported only at baseline; thus, it is impossible to determine whether lower anxiety scores are due to the intervention or to improvements in the underlying cardiac condition. Likewise, not adjusting for concomitant use of psychotropic and anti-arrhythmic drugs prevents conclusions about the effect on cardiac arrhythmias or shocks.

### External Validity

The external validity of the studies was questionable. Most recruiting centres were teaching hospitals or tertiary referral centres. Two of three studies adequately describing the recruitment process reported that only 28% [44] and 26% [46] of the screened population was enrolled in the study, limiting generalizability.

### Limitations of present review

This review has some limitations. First, we included only articles in English published in peer-reviewed journals; studies in other languages might offer different insights. Second, due to the heterogeneous nature of the studies, we only conducted a qualitative analysis.

## Conclusions

Due to methodological limitations, more research is needed to determine whether psychosocial interventions relieve emotional distress in ICD patients. Future studies should be designed as large-scale RCTs, with a longer (1-year) follow up period. Considering the behavioural nature of the interventions, an adequate description of the intervention procedures and an assessment of patients' adherence and providers' treatment fidelity are required. Finally, baseline screening for anxiety and depression would probably allow for the recruitment of patients with significant psychological symptoms that are more likely to benefit from the intervention.

In conclusion, the initial evidence for psychosocial interventions benefiting ICD patients needs to be confirmed by further, methodologically rigorous, research that takes into account the specific challenges of evaluating the effect of a behavioural intervention. Considering the expanding indications for ICD implantation [2,3], the number of ICD candidates and patients experiencing psychological discomfort are destined to increase. Thus, improving their psychological well-being seems worthwhile.

## Competing interests

The authors declare that they have no competing interests.

## Authors' contributions

The first author (ESB) conducted the computer and hand searches, reviewed the abstracts for eligibility together with ISA, performed the data abstraction and drafted the manuscript. The second author (ISA) reviewed the abstracts with ESB and revised the manuscript draft. Both authors read and approved the final manuscript.

## Pre-publication history

The pre-publication history for this paper can be accessed here:

http://www.biomedcentral.com/1471-2261/9/56/prepub

## Supplementary Material

Additional file 1 **Table S1**. Characteristics of included studies Click here for file

Additional file 2 **Table S2**. Main characteristics of the study samples Click here for file

Additional file 3 **Table S3**. Characteristics of study interventions Click here for file

Additional file 4 **Table S4**. Overview of psychological outcomes Click here for file

Additional file 5 **Table S5**. Overview of cardiovascular outcomes Click here for file
