# Supplementary material for: Methodological limitations of psychosocial interventions in patients with an implantable cardioverter-defibrillator (ICD) A systematic review
Source: BMC Cardiovasc Disord. 2009 Dec 29;9:56. doi: 10.1186/1471-2261-9-56 (PMC2809039; doi:10.1186/1471-2261-9-56)
Supplement: Additional file 1 — Table S1. Characteristics of included studies [file 1471-2261-9-56-S1.DOC]

**Table 1** - **Characteristics of included studies**

| **First Author** | **Sample size** | | **Study Location** | **Recruitment duration** | **Study Design** | **Intervention** | **Control** | **Endpoints** | **F.U.**  **(months)** | **Retention rate** |
| --- | --- | --- | --- | --- | --- | --- | --- | --- | --- | --- |
|  | *n*(I) | *n*(C) |  |  |  |  |  |  |  |  |
| Badger 1989  (52) | 6 | 6 | Teaching hospital, US | NR | Quasi-  experimental | Support group | NR | Role functioning, psychological adjustment | 2 | NR |
| Carlsson 2002 (43) | 10 | 10 | Teaching hospital, Sweden | February 1997-April 1998 | RCT | Education | Usual care | QOL | 1 | NR |
| Chevalier 2006 (44) | 35 | 35 | Teaching hospital, France | NR | RCT | CBT | Usual care | Shocks, anxiety, depression, HRV QOL, defibrillator tolerance | 12 | 57% |
| Dougherty  2004, 2005  (41,42) | 84 | 84 | 10 medical centers, US | February 2000-December 2001 | RCT | Phone support | Usual care | Physical functioning, ICD shocks, anxiety, depression, ICD knowledge, health care use | 12 | 89% |
| Edelman 2008  (45) | 13 | 9 | Teaching hospital, Australia | NR | RCT | Education | Usual care | Anxiety, depression, stress, hostility scale | 6 | NR |
| Fitchet 2003  (46) | 8 | 8 | Tertiary referral center, UK | NR | RCT  (cross­over design) | CCR | Usual care | Anxiety, depression shocks, functional status, arrhythmia | 3 | 69% |
| Frizelle 2004 (47) | 12 | 10 | Regional implantation centre, UK | NR | RCT | CCR | Usual care | Anxiety, depression, QOL, ICD concerns, perceived health status, functional capacity | 3 | 95% |
| Kohn 2000  (51) | 25 | 24 | Two teaching hospitals, US | October 1996-July 1997 | RCT | CRT | No therapy | Anxiety, depression, ICD adjustment, sleep, # shocks | 9 | 74% |
| Lewin 2007  (48) | 71 | 121 | Eight ICD implantation centers, UK | February 2004-May 2005 | Cluster RCT | Home-based cognitive-behavioral rehabilitation ("ICD-Plan") | Usual care | Anxiety, depression, ICD shocks, functional status, emergency hospitalizations, cost | 6 | 77% |
| Molchany 1994 (53) | 11 | 5 | NR | NR | Quasi-experimental | Support group | NR | Anxiety, QOL | 9 | NR |
| Sears 2007  (49) | 30  (total sample size) | | Teaching hospital, US | NR | RCT | ICD-SSMP (Education, CBT, relaxation, support group) | One-day workshop | Anxiety, salivary cortisol, QOL, device acceptance, TNFα, IL-6 | 4 | 66% |
| Sneed 1997  (50) | 17 | 17 | Teaching hospital, US | 2 years | RCT | Counseling, phone support, support group | Usual care | Mood states  Psychosocial adjustment | 4 | NR |

*total sample size, n = 30

NR = not reported; CBT = cognitive behavioural therapy; CCR = comprehensive cardiac rehabilitation; F.U. = follow-up; HRV = heart rate variability; n(I) = number of patients in intervention group; n(C) = number of patients in control group; RCT = randomized controlled trial; QOL = quality of life; ICD-SSMP = ICD Shock and Stress Management Program
