# Supplementary material for: Methodological limitations of psychosocial interventions in patients with an implantable cardioverter-defibrillator (ICD) A systematic review
Source: BMC Cardiovasc Disord. 2009 Dec 29;9:56. doi: 10.1186/1471-2261-9-56 (PMC2809039; doi:10.1186/1471-2261-9-56)
Supplement: Additional file 2 — Table S2. Main characteristics of the study samples [file 1471-2261-9-56-S2.DOC]

**Table 2** - **Main characteristics of the study samples**

| **First author** | **Mean age** | **Gender** | **Race/**  **Ethnicity** | **Cardiac**  **history** | **Mean EF (%)** | | **NYHA**  **Class** | | **Timing of enrollment** | **Previous**  **ICD shocks** | | **Anti-Arrhythmics** | **Psycho-tropic**  **drugs** |
| --- | --- | --- | --- | --- | --- | --- | --- | --- | --- | --- | --- | --- | --- |
|  |  |  |  |  | **I** | **C** | **I** | **C** |  | **I** | **C** |  |  |
| Badger 1989  (52) | 48-72  (range) | M/F ratio  I, 6:0  C, 3:3 | NR | NR | 36 | 30 | 1.6  (mean) | 2.0  (mean) | Post -ICD | 12.8 | 6.5 | 2-8 trials | NR |
| Carlsson 2002 (43) | 28-72  (range) | 80% M | NR | 70% “cardiac disease” | NR | | NR | | Pre-ICD | n/a | | NR | NR |
| Chevalier 2006 (44) | 59 | 91% M | NR | CAD (51% I,  48% C) | 36 | 32 | NR | | Pre- and post-ICD | NR | | 60% β-blockers  40% other | NR |
| Dougherty  2004, 2005  (41,42) | 64 | 77% M | 89% white | MI (65% I, 49% C) | 32 | 35 | NR | | Pre-ICD | n/a | | NR | NR |
| Edelman 2008  (45) | NR | 86% M | NR | NR | NR | | NR | | Pre-ICD | n/a | | NR | NR |
| Fitchet 2003  (46) | 58  (34-74) | 88% M | NR | 69% CAD | 41 | | NR | | Post-ICD | NR | | 56% β-blockers | NR |
| Frizelle 2004 (47) | 62 | NR | NR | NR | NR | | NR | | Post-ICD | NR | | NR | NR |
| Kohn 2000  (51) | 66  (42-83) | 65% M | 92% white | NR | NR | | NR | | Pre-ICD | n/a | | NR | NR |
| Lewin 2007  (48) | 61.5 | 83% M | 98%  white | Previous history of PTCA,CABG, pacemaker, angiogram | NR | | 76%  I-II | 75%  I-II | Pre -ICD | n/a | | NR | 15% (I)  14% (C) |
| Molchany 1994 (53) | 51-75  (range) | 87% M | NR | NR | NR | | NR | | Post-ICD | NR | | NR | NR |
| Sears 2007  (49) | 59.8 | 70% M | 77%  white | NR | NR | | NR | | Post-ICD | At least 1 in previous year (eligibility criteria) | | NR | 20% |
| Sneed 1997  (50) | 65 I  61 C | NR | NR | NR | 44 | 34 | NR | | Pre- ICD | n/a | | NR | NR |

NR= not reported; I= intervention, C=control; EF=ejection fraction; NYHA=New York Heart Association; MI=myocardial infarction; CAD= coronary heart disease; PTCA= percutaneous trans-luminal coronary angioplasty; CABG=coronary artery by-pass grafting
