# Supplementary material for: Methodological limitations of psychosocial interventions in patients with an implantable cardioverter-defibrillator (ICD) A systematic review
Source: BMC Cardiovasc Disord. 2009 Dec 29;9:56. doi: 10.1186/1471-2261-9-56 (PMC2809039; doi:10.1186/1471-2261-9-56)
Supplement: Additional file 3 — Table S3. Characteristics of study interventions [file 1471-2261-9-56-S3.DOC]

**Table 3 - Characteristics of study interventions**

| **First author** | **Format and intervention components** | **SO** | **Session duration** | **Duration of intervention** | **Frequency** | **Intervention started** | **Adherence** | **Fidelity assessment** | **Description of usual care intervention** |
| --- | --- | --- | --- | --- | --- | --- | --- | --- | --- |
| Badger 1989  (52) | Group meetings | yes | 1.5 hours | 8 weeks | weekly | Up to12 months after ICD | 87.5% | NR | n/a |
| Carlsson 2002 (43) | Group meetings guided by nurse Educational intervention, phone call after 2 weeks | yes | NR | NR | Before and after implantation | Before ICD | NR | NR | Usual information received from doctor, printed materials |
| Chevalier 2006 (44) | Small groups CBT specialists provided Relaxation tape | no | 2 hours | 12 weeks | bimonthly | NR | NR | NR | NR |
| Dougherty  2004, 2005  (41,42) | Educational (booklets)  Nurse-delivered phone support (scripted) 24/7 access to nurse (pager) | no | 20 minutes | 8 weeks | Once a week | 1 week after ICD | NR | NR | Standard hospital education (booklet and videotape) |
| Edelman 2008  (45) | Group meeting guided by nurse and psychologist; educational (info about ICD) | yes | 1-1.5 hours | 1 session | once | Two weeks after ICD | NR | NR | Usual verbal information form doctor, booklet |
| Fitchet 2003  (46) | Exercise program, educational seminars, psychologist-provided CBT, w/ individual support if high scores for anxiety, relaxation techniques (self-hypnosis, re-breathing) | yes | 2 hours | 12 weeks | Twice a week | Two weeks after ICD | NR | NR | Access to educational help line, ICD technician, support group |
| Frizelle 2004 (47) | CBT; relaxation techniques (tapes), breathing retraining; home-based exercise, program; follow-up phone call | no | 2 hours | 6 weeks | Once a week | NR | NR | NR | wait-list (routine visits to clinic) |
| Kohn 2000  (51) | One- to one CBT administered by doctoral level psychology student (over the phone when meetings not possible) | yes | 1/2-1 hour pre-ICD; 15'- l/2 hour after | 20 weeks | 4 weekly sessions, shorter CBT at 4, 12, 20 weeks | Before ICD implant | NR | NR | n/a |
| Lewin 2007  (48) | Self-administered educational and CBT  intervention, relaxation techniques (booklets, tapes/CD), phone support | no | n/a | NR | NR | Immediately before  ICD | NR | NR | Education about procedure, booklet, phone call from facilitator |
| Molchany 1994 (53) | Group meetings led by psychiatric nurse specialist (no script/description) | yes | 1.5 hours | NR | weekly | Post-ICD (5-6 months from ICD for 55% of pts, 12-24 months for 45%) | NR | NR | n/a |
| Sears 2007  (49) | Group meetings, ICD-specific education. relaxation/stress-management training.  CBT, group discussion and social support | no | 1 .5 hours | 6 weeks | weekly | NR | NR | NR | n/a |
| Sneed 1997  (50) | In-hospital counseling, relaxation techniques, ICD support meetings  Phone calls | yes | NR | 16 weeks | Twice total  weekly until week 8, then bimonthly | During hospitalization for ICD | NR | NR | NR |

SO=significant others; NR= not reported; CBT= cognitive behavioural therapy
