# Supplementary material for: Methodological limitations of psychosocial interventions in patients with an implantable cardioverter-defibrillator (ICD) A systematic review
Source: BMC Cardiovasc Disord. 2009 Dec 29;9:56. doi: 10.1186/1471-2261-9-56 (PMC2809039; doi:10.1186/1471-2261-9-56)
Supplement: Additional file 4 — Table S4. Overview of psychological outcomes [file 1471-2261-9-56-S4.DOC]

**Table 4 Overview of psychological outcomes**

| **First Author** | **Psychological outcome** | **Instrument used** | **Overall effect** |
| --- | --- | --- | --- |
| Badger 1989  (52) | Psychological adjustment | RSFHS | Non-significant improvement of pre intervention RSFHS scores in intervention group (19.6±7.3) vs. post (21.1 ±7.6), no change in control group |
| Carlsson 2002 (43) | - | - | - |
| Chevalier 2006 (44) | Anxiety | HAMA | Reduction in mean scores at 3 months (6.15±6.74 to 3.23±2.55) and 12 months (2.62±1.45) in intervention vs. control, p<0.05* |
|  | Depression | BDI | Non-significant trend in scores reduction (p=0.07)* |
| Dougherty  2004, 2005  (41,42) | Anxiety | STAI | Mean STAI scores in intervention: baseline, 36.1±10.7; month: 33.7±10.8, 3 months: 31.9 ±11.7  Control: 33.1±10.7, 32.1±10.3, 33.0±11.2; p=0.49 and 0.08. At 12 months, p<0.01§ |
|  | Depression | CES-D | No difference |
|  | Mental health | SF-12 | No difference |
| Edelman 2008  (45) | Anxiety  Depression  Stress | DASS | No association between any of the psychological outcomes and the intervention, adjusted for age and gender |
| Fitchet 2003  (46) | Anxiety | HADS | Significant decreases in mean scores for anxiety from pre (13.4±3.6) to post- intervention (8.1±3.6), p<0.001, maintained at 12 weeks (6.1±4.5)¶ |
|  | Depression | HADS | Significant reduction in depression scores |
| Frizelle 2004 (47) | Anxiety  Depression | HADS | Increase in the difference between pre-post mean HADS scores for anxiety (-1.08 treatment vs. 0.10 waiting list; p = 0.012) and depression (-1.58 vs. -0.10, p=0.001 |
| Kohn 2000  (51) | Anxiety | STAI | At 9 months lower mean trait-anxiety scores in intervention group (30.9±9.9 vs. 41.1±13.3, p=0.013)  No difference in anxiety scores |
|  | Depression | BDI | Decrease in mean depression scores in CBT group^#^ |
| Lewin 2007  (48) | Anxiety | HADS | No difference in mean HADS scores between groups at six months  Reduction in proportion of patients with co-morbid anxiety in intervention (-21%) vs. control group (-13%)  P value not reported $ |
|  | Depression | HADS | Reduction in proportion of patients with co-morbid depression (-13% vs. -2.1%). P value not reported.  No difference in mean HADS scores in intervention and control group at 6 months |
|  | Mental health | SF-12 | Non-significant reduction in mean scores differences between groups |
| Molchany 1994 (53) | Anxiety | STAI | No difference in pre-post treatment mean scores for anxiety (Pre: 36±11.7 vs. post: 34.8±11.3, n.s.) |
|  |  | anxiety VAS | No difference in pre-post treatment mean VAS scores: (20.6 ±17 vs. 34.2±29.3, n.s.) |
| Sears 2007  (49) | Anxiety | STAI | Reduction in pre- (37.8 ± 9.2) vs. post-treatment (32.1.± 5.1, p=0.001) mean scores (time effects, whole sample). Significant difference between intervention (pre: 41.7 ±9.9, post 32.8±4.5) and control, pre: 33.4±6.1 post: 31.3±5.5, p=0.03 for group by time interaction). No changes at 4 months F.U. |
|  | Depression | CES-D | Reduction in pre-post treatment mean scores in both groups, no difference between groups. At 4 months increase in depression scores in control group (p = 0.02). |
|  | Mental health | SF-12 | Increase in pre-post treatment mean scores, significantly higher in intervention group. No changes at 4 months F.U. |
| Sneed 1997  (50) | Mood states | POMS | No differences between mean POMS scores between intervention (33.1±51.2) and control (19.5±32.4) at 4 months of F.U. (adjusted for baseline scores). |

RSFHS = Rand Short-Form Health Survey; POMS = profile of Mood States; HADS = Hospital Anxiety and Depression Scale; STAI = State-Trait Anxiety Inventory; CES-D= Center for Epidemiological Studies Depression Scale; BDI= Beck Depression Inventory; HAMA= Hamilton Anxiety Scale; DASS= Depression Anxiety Stress Scale; VAS= visual analogue scale. F.U.= follow-up

*only 13 I, 16 C subjects available for analysis; SD are different in tables and results section. Within-group analysis.

§ adjusted for age gender, co-morbidities, SES, ICD indication. Between-group comparisons. At 12 months means not reported.

¶ n=11, within group analysis.

# No baseline depression scores, no adjustment for baseline scores.

$ adjusted for baseline HADS scores, and clustering effects.
