# Supplementary material for: Methodological limitations of psychosocial interventions in patients with an implantable cardioverter-defibrillator (ICD) A systematic review
Source: BMC Cardiovasc Disord. 2009 Dec 29;9:56. doi: 10.1186/1471-2261-9-56 (PMC2809039; doi:10.1186/1471-2261-9-56)
Supplement: Additional file 5 — Table S5. Overview of cardiovascular outcomes [file 1471-2261-9-56-S5.DOC]

**Table 5 - Overview of cardiovascular outcomes**

| **Authors (Reference)** | **Shocks/ATP** | **HRV** | **Hospitalizations** | **Exercise capacity** |
| --- | --- | --- | --- | --- |
| Badger 1989  (52) | **_** | **_** | **_** | **_** |
| Carlsson 2002 (43) | _ | _ | _ | _ |
| Chevalier 2006 (44) | At 12 months 3 patients had shocks in CBT vs. 6 in usual care  Reduction in shock rate; reduction in use of beta-blockers and AA (post-hoc) | Improved adrenergic/vagal balance in CBT vs. usual care | **_** | **_** |
| Dougherty  2004, 2005  (41,42) | No differences at 1, 3, 6 and 12 months. | **_** | No difference in mean ER visits, hospital admissions, clinic visits between groups | **_** |
| Edelman 2008  (45) | **_** | **_** | **_** | **_** |
| Fitchet 2003  (46) | No change in shocks frequency, VT requiring ATP, non-sustained VT. | _ | _ | % Increase in pre/post intervention exercise time, maintained at 3 months post-intervention (treadmill) |
| Frizelle 2004 (47) | No change in # of shocks or ATP | **_** | **_** | Significant increase in level of difficulty and distance walked (Shuttle) |
| Kohn 2000  (51) | No differences in mean numbers of shocks between groups | **_** | **_** | **_** |
| Lewin 2007  (48) | Non-significant reduction in proportion of patients experiencing shocks* | **_** | Significant reduction in mean number of emergency admissions* | Significant improvement in Seattle angina questionnaire-physical limitations score (self-administered questionnaire)* |
| Molchany 1994 (53) | **_** | **_** | **_** | **_** |
| Sears 2007  (49) | **_** | **_** | **_** | **_** |
| Sneed 1997  (50) | **_** | **_** | **_** | **_** |

AA=anti-arrhythmics; CBT=cognitive behavioral therapy; ER= emergency room; ATP= anti-tachycardia pacing; HRV= Heart rate variability; VT= ventricular tachycardia.

* Adjusted for clustering
